# Redispersible Acrylic Ester Polymers: Effect of Polymer Property Changes Due to Polymerization Method Modification and Functional Additives on the Performance of Polymer Cement Mortar

**DOI:** 10.3390/ma17225534

**Published:** 2024-11-13

**Authors:** Jeong-Bae Lee

**Affiliations:** Department of Smart Construction and Environmental Engineering, Daejin University, Pochon 11159, Republic of Korea; jblee@daejin.ac.kr

**Keywords:** emulsion monomer dropping, monomer dropping, average particle size, cement mortar

## Abstract

This paper presents an experimental study aimed at improving the performance of polymer cement mortar by evaluating the properties of acrylic ester redispersible polymers, synthesized using a change in polymerization method from emulsion monomer to monomer dropwise addition methods, along with the use of a functional additive in the form of a foaming agent. To achieve the research objectives, a polymer with a glass transition temperature of −11 °C was synthesized by fixing the monomer ratio, particle-size distribution, and glass transition temperature, and the physical properties of the polymer cement mortar were assessed. The results showed that polymers synthesized using the modified polymerization method increased elongation at break and possessed a 35% smaller average particle size. The use of the foaming agent also resulted in enhanced tensile strength. The polymer cement mortars made with these respective polymers demonstrated improvements in compressive strength 11~25%, flexural strength 53~77%, bond strength 78~113%, volumetric changes 65~88%, and water absorption 30~70%. These findings suggest that changes in the polymerization method and the incorporation of functional additives influence the average particle size and air entrainment control properties of the polymers, thereby positively impacting the performance of the cement hydrates.

## 1. Introduction and Motivation

Cement mortar and concrete, widely used as repair materials for concrete structures, are convenient to use and inexpensive but have disadvantages such as low tensile and flexural strength, high drying shrinkage, low bond strength, and poor chemical and freeze–thaw resistance. To overcome these weaknesses, polymers are incorporated into cement mortar and concrete.

The use of polymers in cement mortar and concrete was first introduced by Cresson in 1923, and the research on the polymer forms currently in use was published by Lefebure in 1924. Since then, significant research and development has been conducted to utilize polymers in mortar and concrete, leading to the production of polymer-modified materials in several countries for the last 70 years [1]. During the 1920s and 1930s, polymer-modified cement mortar and concrete were developed using natural rubber latex, and in the 1940s, research on synthetic latex, chloroprene rubber latex, and polyacrylic ester latex for polymer-modified cement mortars began. By the late 1940s, these materials had seen diverse applications. In the 1960s, the use of styrene-butadiene rubber, polyacrylic ester, and polyvinylidene chloride-vinyl chloride in mortar and concrete became more widespread. This led to the advancement of polymer-modified cement mortar and concrete in several countries. Additionally, methylcellulose, a water-soluble polymer, was applied to polymer-modified cement mortar for ceramic tile adhesives [2].

Shibazaki introduced research on polymer-modified cement mortars using hydrophilic polymers such as hydroxyethyl cellulose and polyvinyl alcohol [3]. Shiyun and Zhiyuan studied polymer-modified cement mortar using composite polymer latexes which showed that mixing two different polymer latexes can complement the disadvantages of each polymer [4].

In 1933, Sugita et al. researched the microstructure and the complex mechanism of latex paste and mortar, discovering that a large amount of polymer particles and hydrated cement form layers through interfacial action within the aggregate and cement particles [5]. Geist, who developed PVAC mortar, found that particle sizes between 1 and 5 μm or 2 and 5 μm produce maximum strength [6]. In 1964, Ohama et al. studied the monomer ratios of EVA, SBR, and poly styrene-butyl acryl ester, concluding that the monomer ratio affects the strength of latex mortars within the same range as the polymer–cement ratio [7]. In 1987, they introduced a mechanism involving interactions between the polymer and carboxyl groups [8].

Today, the construction industry demands high-performance materials for improving the durability of structures and applying advanced construction methods. Polymer-modified cement mortar and concrete are receiving attention in this context. In general, polymer-modified cement mortar is a composite material consisting of cement and polymers mixed for cement modifications. Compared to standard cement mortar, it demonstrates superior workability, repairability, flexural and tensile strength, flexural toughness, adhesion, waterproofing, resistance to chloride ion penetration, resistance to carbonation, freeze–thaw resistance, abrasion resistance, and impact resistance [9,10]. Due to these advantages, polymer-modified cement mortars are widely used around the world in high-performance building exterior materials, flooring, coatings, waterproofing materials, decorative coatings, and for repairing and reinforcing reinforced concrete structures [11].

The characteristics of polymer-modified cement mortar and concrete are affected by the properties of the polymer film formed inside the mortar. Thus, the polymer film formation process is closely related to the properties of polymer-modified cement mortar and concrete. Understanding the inherent properties of polymers, such as the strength and elongation of the polymer film, the minimum film formation temperature (MFT), the glass transition temperature (Tg), the melting point (Tm), and the lower temperature limits, is crucial before applying them to cement mortar and concrete. Among these, Tg is a key factor in determining the degree and form of film formation within cement mortar and concrete [12].

Since the 2010s, research utilizing natural polymers such as starch, cactus extract, casein, rice, and eggs has reported that polymer incorporation affects the durability and waterproofing of mortar [13,14]. Research on the fire resistance of polymer-modified mortars has also been conducted, evaluating characteristics such as heat resistance, residual bond strength, and tensile strength [15,16,17]. Although various studies on polymer-modified mortars have been carried out, there is still a lack of research focused on improving the performance of polymer-modified cement mortar by modifying polymer properties through synthesis processes.

This paper evaluates the improvement in the performance of polymer-modified cement mortars using polymers with a glass transition temperature of −11 °C, which were identified as suitable for cement modifications in previous studies. These polymers were modified through changes in synthesis methods and the use of additives, and their effects on the performance of cement pastes were studied.

## 2. Research Objectives and Scope

In this paper, the glass transition temperature used was −11 °C (AC3), which demonstrated the best overall performance in previous research [18] evaluating the physical properties of acrylic-based redispersible powder polymers synthesized with varying monomer ratios. Based on the selected monomer ratio, the characteristics of polymer-modified cement mortars were evaluated using polymers whose performance was modified through two methods.

There are several methods to improve the performance of polymers used in cement mixtures. Common approaches include adjusting the hardness through molecular weight control, adding functionality through functional monomers, and using additives such as dispersants and thickeners. These methods allow for the modification of polymer properties to achieve the most suitable characteristics for specific applications.

This research employed two methods: using a defoamer as a functional additive to control the entrained air within the polymer and modifying the polymerization method; specifically, the polymerization method from emulsion monomer to monomer dropping systems to reduce the polymer particle size and achieve stable synthesis. Additionally, modified polyvinyl alcohol was replaced with polyvinyl alcohol, which is used as protective colloid, to further enhance the performance of the polymer.

The research content and methods of this paper are as follows:

(1) Polymers were synthesized based on a glass transition temperature of −11 °C (AC3) according to the monomer ratio. Two types of polymers were synthesized: a polymer modified with a defoamer as a functional additive (defoamer-modified acrylic latex; DMAC) and a polymer synthesized by changing the emulsion polymerization method and protective colloid (polymerization-modified acrylic latex; PMAC). The basic properties of these redispersible powder polymers were evaluated.
(2) The glass transition temperature was measured using a differential scanning calorimeter, and the tensile strength was measured according to KS M 3381 (testing method for tensile properties of glass-fiber-reinforced plastics) [19]. Additionally, the average particle size was analyzed using a particle-size analyzer.
(3) Polymer-modified cement mortar was prepared using the synthesized redispersible powder polymers. The polymer-modified cement mortar was made by incorporating 5% of the total binder with redispersible powder polymer, while maintaining an air content of 4.5 ± 1.5% and a flow value of 155 ± 10 mm to ensure consistent workability. The setting time, compressive strength, flexural strength, bond strength, length change, and water absorption of the polymer-modified cement mortars were measured at various curing ages and compared with standard cement mortar and a polymer-modified cement mortar before performance modification.

## 3. Overview of Experiments

### 3.1. Materials Used

The cement used in this study was Type 1 Ordinary Portland Cement, as specified in KS L 5201 [20]; its chemical composition and physical properties are shown in Table 1. The physical properties of the standard sand are also shown in Table 1 and Table 2.

Additionally, the physical properties of the silica fume (hereafter referred to as SF) used in this study are shown in Table 3. A melamine-based powdered superplasticizer was used in the preparation of both cement mortar and polymer-modified cement mortar.

### 3.2. Experimental Mix Proportion

#### 3.2.1. Polymer Synthesis

The acrylic ester copolymer emulsion composition used in this study consisted of a hydrophilic ethylene-unsaturated monomer and a hydrophobic ethylene-unsaturated monomer. Methyl (meth)acrylate (MMA) was used as the hydrophilic ethylene-unsaturated monomer, while butyl (meth)acrylate (BA), ethyl acrylate (EA), and 2-ethylhexyl acrylate (2-EHA) were used as the hydrophobic ethylene-unsaturated monomers, with the monomer ratios adjusted accordingly. The glass transition temperature (Tg) of the synthesized emulsion was fixed at −11 °C. The compositions of the reference polymer AC3 and the performance-modified polymers DMAC and PMAC are shown in Table 4.

#### 3.2.2. Polymer Cement Mortar

The mix proportions of the polymer cement mortar used in this study are shown in Table 5.

### 3.3. Experimental Method

#### 3.3.1. Performance of Redispersible Powder Polymer

(1) Glass transition temperature

The thermal properties, specifically the glass transition temperature (Tg), of the polymers synthesized with the monomer ratios shown in Table 4 were measured. To measure Tg, a portion of the polymer film, which was dried in an oven, was sampled and scanned using differential scanning calorimetry (DSC) at a rate of 10 °C/min over a range of −50 °C to 50 °C. The theoretical calculation of Tg without experimental testing is shown in Equations (1)–(4):

T_wt_ = (T_wt_1 + T_wt_2 + T_wt_3 + … + T_wt_n) (1)

T_gt_ = (T_g_1 + T_g_2 + T_g_3 +… + T_g_n) (2)

T_g_1, T_g_2, T_g_3, …, T_g_n = (K + T_gm_) (3)

T_wt_/T_g_ = {(T_wt_1/T_g_1) + (T_wt_2/T_g_2) + (T_wt_3/T_g_3) + … + (T_wt_n/T_g_n)} (4)

where:

T_wt_: Total weight of the monomer
T_gt_: Total glass transition temperature (sum of the glass transition temperature of individual monomers and absolute temperature)
T_gm_: Glass transition temperature of the monomers used as raw materials
K: Absolute temperature

(2) Tensile Strength

To evaluate the properties of the synthesized polymer, specimens were prepared by drying in an oven at 40 °C for 48 h. The tensile strength and elongation were measured according to KS M 3381, “Test Method for Strength of Glass Fiber Reinforced Plastics” [19]. Each powdered polymer was redispersed by diluting 20% in water, then molded on a glass plate and dried to form films. These films were cut into specimens of 10 mm × 30 mm and tested using a tensile-strength-measuring device.

(3) Average Particle Size

To analyze the particle size of the synthesized redispersible polymer, the average particle size of the powder polymer was measured using a particle-size analyzer.

(4) Microstructure Analysis

To observe the morphology of polymer films formed inside the cement paste, analyses were conducted using scanning electron microscopy (SEM) and energy dispersive X-ray spectroscopy (EDS).

#### 3.3.2. Performance of Polymer Cement Mortar

(1) Setting Time

To determine the setting characteristics of the polymer cement mortar, the setting time of the mortar was measured according to KS F2436, “Test Method for Setting Time of Concrete by Penetration Resistance” [21].

(2) Flow and Air Content

To measure the flow and air content of the polymer cement mortar, the flow and air content of the mortar were kept constant. The flow of the mortar was measured using a flow table according to KS F 2476, “Test Method for Polymer Cement Mortar”, [22] after preparing the mortar. The air content was measured according to KS L 3136, “Test Method for Measuring the Air Content of Hydraulic Cement Mortar” [23].

(3) Compressive Strength

To measure the compressive strength of the polymer cement mortar, 50 × 50 × 50 mm cube molds were used to prepare the specimens. After curing for 48 hours at a temperature of 20 ± 2 °C and relative humidity above 90%, the specimens were demolded and water-cured for 5 days at 20 ± 2 °C. Then, they were cured at 20 ± 2 °C and 60 ± 10% relative humidity for 21 days. The compressive strength was measured on days 3, 7, 28, and 56 according to the test method specified in KS L 5105 [24].

f_m_ = P/A (5)

where:

f_m_: Compressive Strength (Mpa)
P: Total maximum load (N)
A: Area of loaded surface (mm^2^)

(4) Flexural Strength

To measure the flexural strength of the polymer cement mortar, 40 × 40 × 160 mm prismatic molds were used to prepare the specimens. After molding, the specimens were cured in the same manner as the compressive strength specimens. The flexural strength was measured on day 28 according to the test method specified in KS F 2476 [22].

S_f_ = 0.0028P (6)

where:

S_f_: Flexural Strength (Mpa)
P: Total maximum load (N)

(5) Bond Strength

To measure the bond strength of the polymer cement mortar, a base concrete specimen (600 × 600 × 100 mm) with a compressive strength of approximately 50 MPa was prepared. Mortar was applied to the specimen at a thickness of 5 mm. Bond strength was measured on days 7, 14, and 28 by attaching a 40 × 40 mm top tensile jig, following the bond strength test methods specified in KS F 4715 [25], JIS A 6909 [26], and JIS A 6910 [27] (thin coating materials).

S_t_ = P/A (7)

where:

f_m_: Bond Strength (Mpa)
P: Total maximum load (N)
A: Area of loaded specimen (mm^2^)

(6) Volume Change

To measure the volume change of the polymer cement mortar, the length change of each polymer cement mortar was compared to that of standard cement mortar. A 25 × 25 × 285 mm prismatic mortar specimen for length change was prepared and cured in the same way as the compressive strength specimens. The length change was measured over 56 days at 20 ± 2 °C and 60 ± 10% relative humidity using a dial gauge method according to KS F 2424, “Test Method for Length Change of Mortar and Concrete” [28] and calculated as per Equation (5).

Lt = {(L_0_ − L_n_)/L_0_} × 100 (8)

where:

L_t_: Length change rate (%)
L_0_: Length of the mortar before immersion
L_n_: Length of the mortar at n days of immersion

(7) Water Absorption

To evaluate the resistance of the polymer cement mortar to water absorption, a water absorption test was conducted in accordance with KS F 2476, “Test Method for Polymer Cement Mortar” [22].

The polymer cement mortar specimens were prepared based on each mix proportion and cured for 28 days. After curing, the specimens were dried in an oven at 80 ± 2 °C for 24 h. The initial weight of the dried specimens was measured, and the specimens were immersed in water at 20 ± 2 °C. The weight of the specimens was measured at 1, 3, 5, 9, 24, and 48 h of immersion. The water absorption rate was calculated using Equation (6).

(9) $$W_{a}=\frac{W_{1}-W_{0}}{W0}\times 100$$

W_a_: Water absorption rate (%)
W_1_: Weight of the specimen at each immersion time (g)
W_2_: Weight of the specimen before immersion (g)

## 4. Results of Experiments

In this study, redispersible powder polymers were synthesized based on the monomer ratio of AC3, which has a glass transition temperature of −11 °C. Two types of polymers were produced: one combined with a functional additive and another with modified protective colloid and polymerization method. The functional additive used was a defoamer, which aimed to prevent performance degradation caused by excessive entrained air in the redispersible powder polymer. Additionally, modified polyvinyl alcohol (PVOH) was used as the protective colloid, and the polymerization method was changed from the emulsion monomer dropping system to the monomer dropping system.

### 4.1. Physical Properties of Polymers

The redispersible powder polymers were analyzed for melting point, glass transition temperature (Tg), tensile strength, and particle-size distribution. The glass transition temperature was measured using differential scanning calorimetry (DSC), and the tensile strength and elongation were measured using a tensile strength tester (AGS-500G, Shimadzu, Kyoto, Japan). To analyze the particle size of PMAC and AC3, a particle-size analyzer was used to determine the average particle size of the powder polymers.

**Figure 1:**
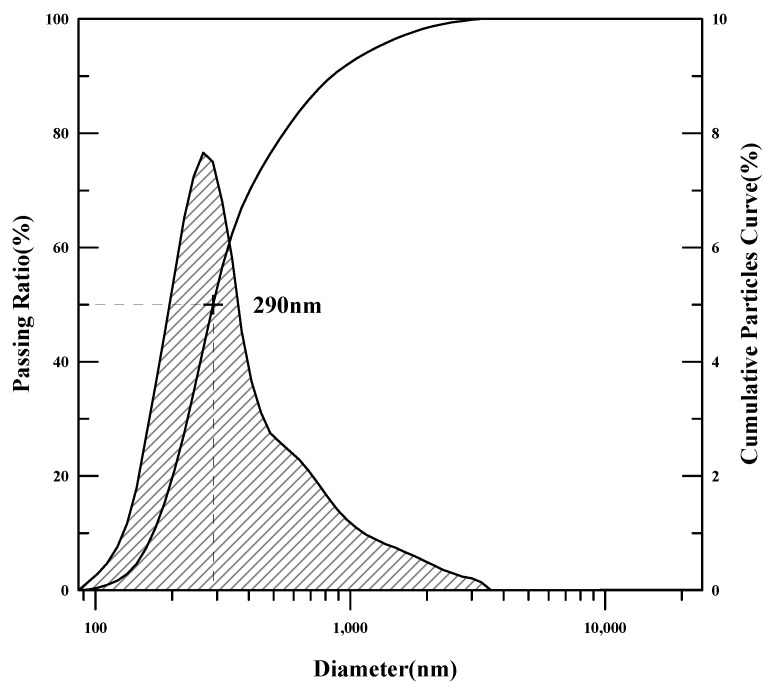
Particle-size analysis results (PMAC).

**Figure 2:**
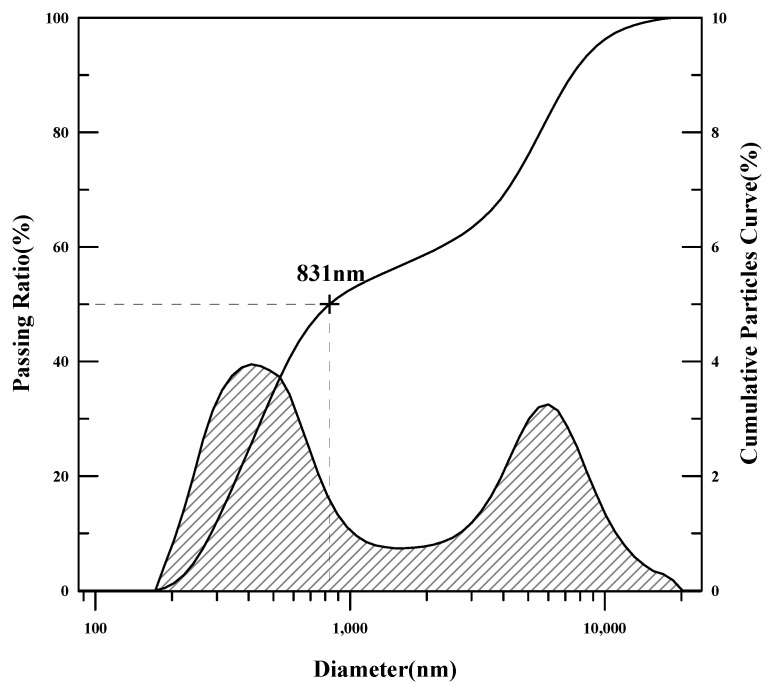
Particle-size analysis results (AC3).

**Figure 3:**
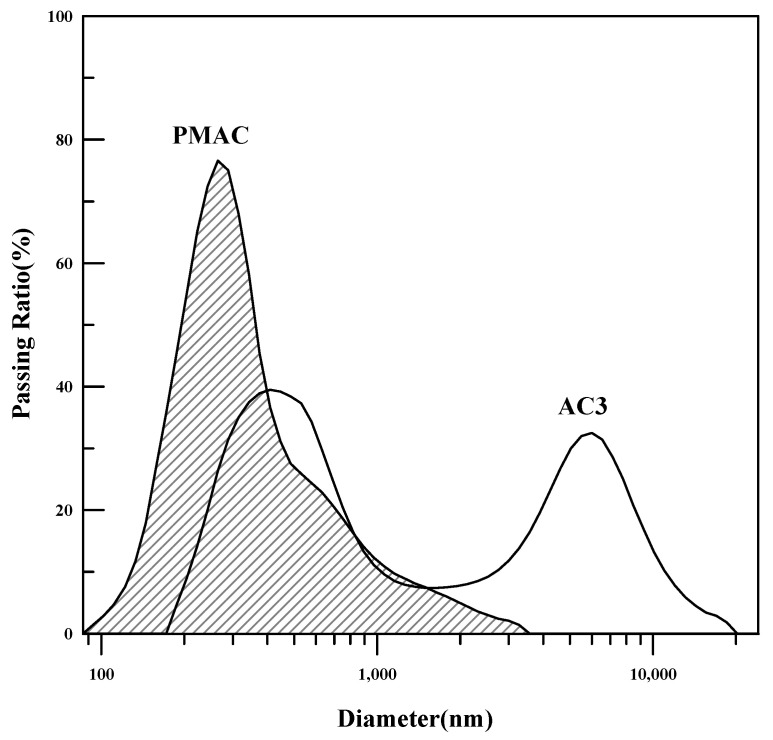
Changes in particle-size distribution due to polymerization method modification.

**Figure 4:**
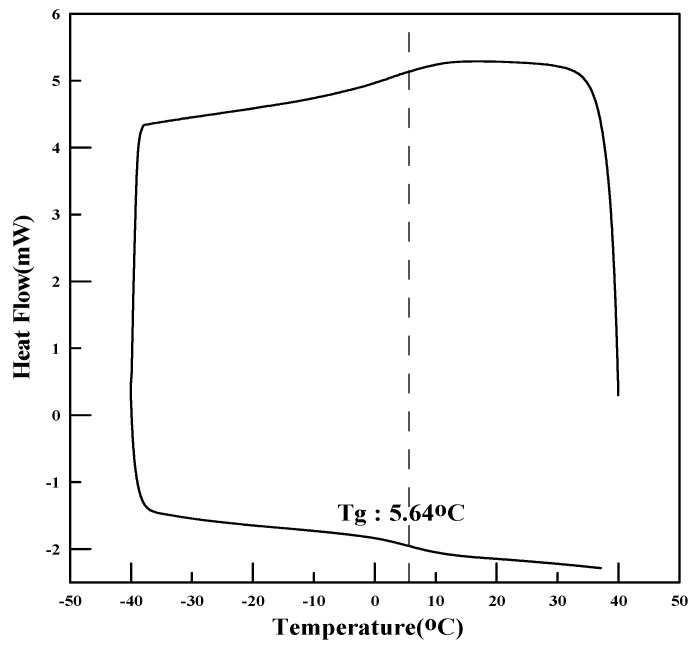
DSC measurement results (DMAC).

**Figure 5:**
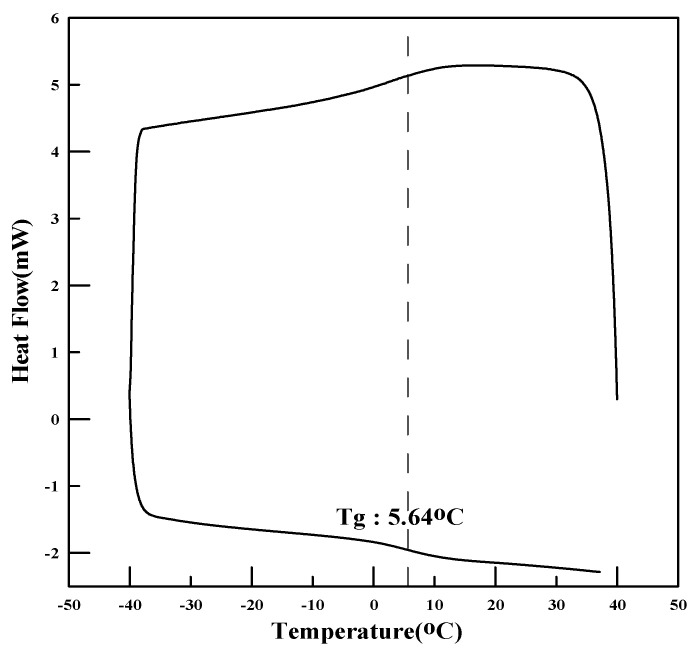
DSC measurement results (PMAC).

**Figure 6:**
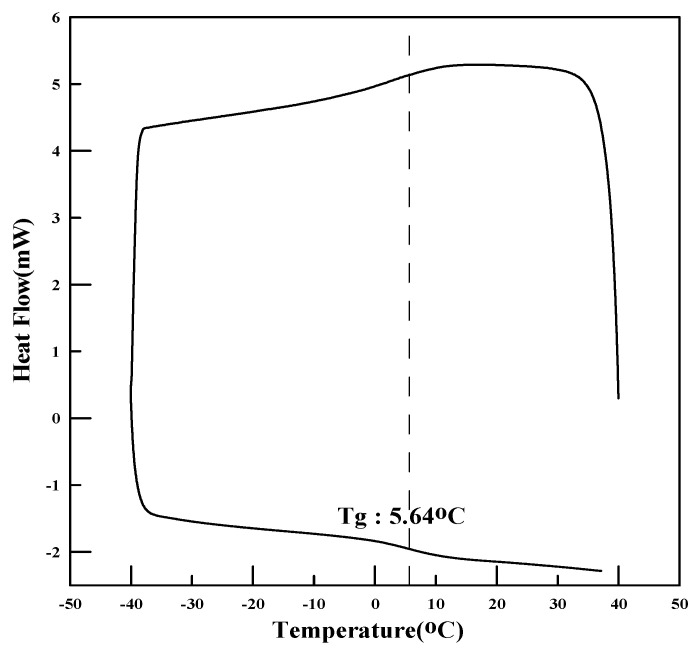
DSC measurement results (AC3).

The physical properties of the polymers are presented in Table 6 and Figure 1, Figure 2, Figure 3, Figure 4, Figure 5 and Figure 6. As the analysis results show, the theoretical Tg remained the same since there was no change in the monomer ratio. Additionally, the modification of the polymerization method and the use of a defoamer did not affect the glass transition temperature. The Tg values measured using DSC were approximately 10 °C higher than the theoretical Tg. This discrepancy is due to the fact that the theoretical Tg is calculated based on the inherent glass transition temperature of each monomer, which matches the measured value when the polymer is in its emulsion state. However, the presence of this anti-caking agent, which is applied onto the surface of the powdered polymer particles to prevent them from clumping and to ensure long-term preservation, causes the Tg of the redispersible powder resin to be higher than the theoretical Tg when analyzed using DSC. Once redispersed, the polymer behaves according to its original Tg characteristics (18).

In terms of tensile strength, DMAC indicated a slightly higher value. Furthermore, the film elongation of PMAC, which was synthesized using a modified polymerization method, was 46% higher.

The average particle-size analysis revealed that the particles of PMAC, synthesized with the modified polymerization method, were 35% smaller than those of AC3. Since DMAC was synthesized using the same polymerization method as AC3, the average particle size was the same. The increase in tensile strength and slight decrease in elongation for DMAC are likely due to the suppression of air bubbles within the polymer, which resulted in stronger films. In the case of PMAC, the significant reduction in average particle size is attributed to the characteristics of the monomer dropping system and the modified protective colloid.

These results suggest that the use of modified polymers could lead to improved performance in polymer cement mortars.

### 4.2. Physical Properties of Polymer Cement Mortar

#### 4.2.1. Flowability and Setting Characteristics

(1) Flowability

Table 7 summarizes the air content and flow values for the polymer cement mortar and standard cement mortar, which were prepared using AC3, DMAC, and PMAC, organized by polymer type.

As shown in the results of the experiment, the air content of DMAC, which includes a defoamer, was reduced by approximately 10% compared to AC3 (before the addition of the defoamer). This indicates that the addition of a defoamer during the polymerization stage effectively controls entrained air. In this study, a defoamer was added after polymerization to maintain a consistent air content, but post-addition alone was not able to control the entrained air. Therefore, the addition of a defoamer during the polymerization stage is considered to be a more effective method. This is because controlling the entrained air reduces the pore volume inside the cement mortar and increases the number of fine pores, thereby improving compressive strength.

In the case of PMAC, compared to AC3, there was a slight decrease in flow and a small increase in air content. However, overall, PMAC had similar behavior to AC3.

Generally, an increase in air content tends to enhance flowability, but in polymer cement mortar using PMAC, the flowability did not increase despite the rise in air content. It is assumed that the particle size of PMAC, produced with a modified polymerization method, was smaller than that of AC3, leading to an increase in the specific surface area of the polymer in contact with water within the polymer cement mortar. In other words, the increase in fine particles resulted in higher viscosity of the polymer cement mortar, leading to reduced flowability.

(2) Setting Characteristics

**Figure 7:**
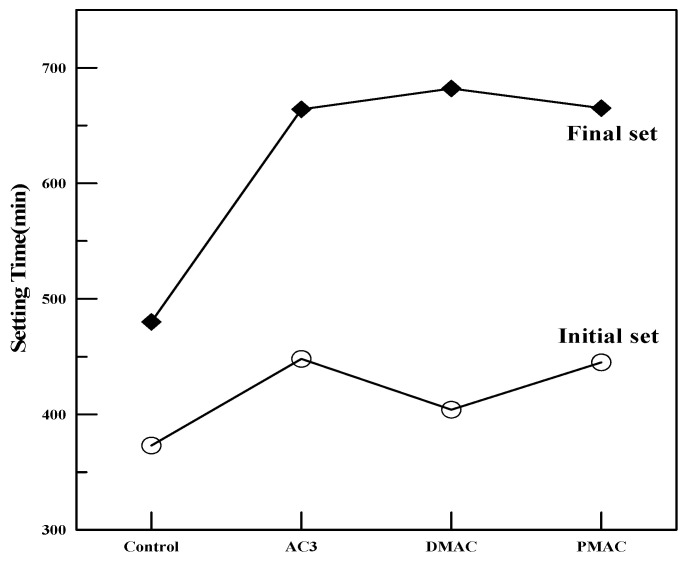
Changes in setting time of mortar due to polymer performance modifications.

To compare the setting characteristics of polymer cement mortar using DMAC, PMAC, and AC3 with ordinary cement mortar, the setting time of the mortar was measured according to KS F 2436, “Test Method for Setting Time of Concrete by Penetration Resistance” [21]. The results of the setting time test are summarized by polymer type in Figure 7.

As seen in Figure 7, polymer cement mortar tends to have a delayed setting time compared to ordinary cement mortar.

Additionally, there was no significant difference in the setting time between AC3, which used the original polymer, and PMAC, which used the modified polymer. This is likely because both polymers had the same glass transition temperature. However, DMAC showed a slightly faster initial setting compared to AC3 and PMAC. This trend is attributed to the defoamer added during the polymerization process, which controlled the entrained air in the polymer, leading to a reduction in air voids within the polymer cement mortar. The reduction in internal voids increased the penetration resistance, as the voids were reduced, hindering the penetration of the needle, thus resulting in higher penetration resistance values.

In the case of PMAC, the initial setting time was delayed compared to AC3, but the final setting time occurred earlier, showing the same trend compared to DMAC. This can be explained by the smaller polymer particle size, which increases the number of polymer particles surrounding the cement particles, thereby hindering the hydration of water and cement, delaying the initial setting. Additionally, the faster redispersible rate of the finer polymer particles in water accelerated the formation of polymer films within the polymer cement mortar, increasing the penetration resistance and causing the final setting to occur earlier.

#### 4.2.2. Strength Characteristics

(1) Compressive Strength

The compressive strength of polymer cement mortar and ordinary cement mortar prepared with the three types of powder polymers was measured at 3, 7, 28, and 56 days of curing. The results are summarized in Table 8.

**Figure 8:**
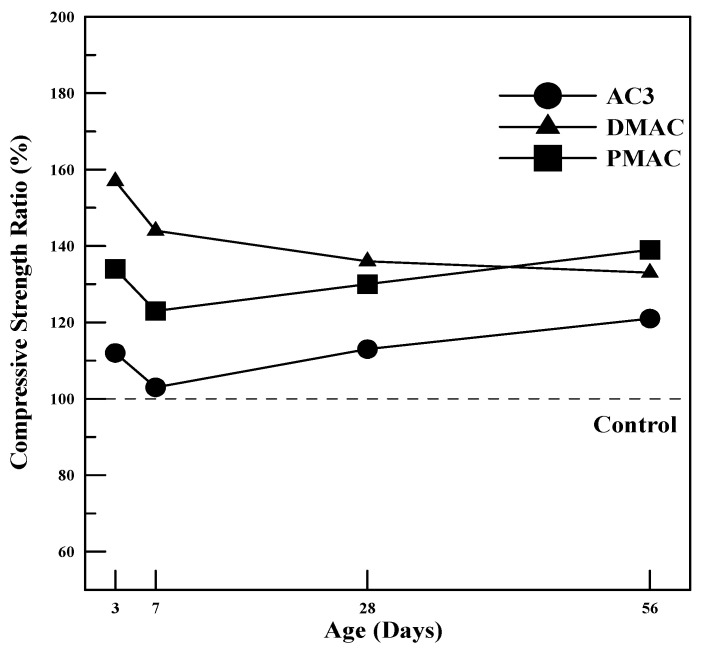
Compressive strength ratio of polymer cement mortar.

To evaluate the compressive strength development characteristics of polymer cement mortar using the three types of powder polymers, the compressive strength ratio of polymer cement mortar to cement mortar at each curing age is shown in Figure 8. As illustrated in Figure 8, both DMAC and PMAC had a 20–40% increase in compressive strength compared to AC3. Furthermore, compared to ordinary cement mortar, the compressive strength increased by approximately 30–50%, indicating that the use of modified polymers effectively enhances the strength of polymer cement mortar.

When comparing DMAC and PMAC, which utilize different methods for performance improvement, slightly different trends were observed. DMAC showed the best compressive strength results up to 28 days, while PMAC indicated the highest strength at 56 days. These results suggest that the method of controlling entrained air to improve performance is particularly effective in enhancing early strength. On the other hand, in the case of improving performance by modifying the emulsion polymerization method, the reduction in polymer particle size accelerated the formation of polymer films within the polymer cement mortar. This led to the creation of denser films, which suppressed moisture evaporation inside the mortar and allowed the hydration process to continue over an extended period. As a result, strength tends to increase over time with greater gains at longer curing ages compared to the early stages.

(2) Flexural Strength

Table 9 summarizes the flexural strength at 28 and 56 days for polymer cement mortars prepared using AC3, DMAC, and PMAC, along with the flexural strength ratio of polymer cement mortar compared to ordinary cement mortar at the same curing ages.

**Figure 9:**
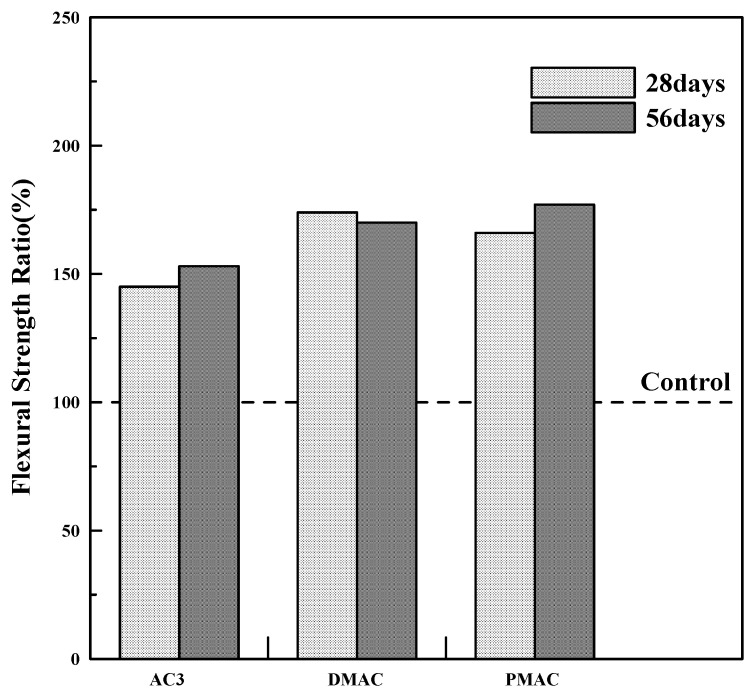
Flexural strength ratio of polymer cement mortar.

To examine the flexural strength development characteristics of polymer cement mortar before and after performance modification, the flexural strength ratio of the specimens at 28 and 56 days is summarized in Figure 9. As shown in Figure 9, DMAC and PMAC had a 15–20% increase in flexural strength at 28 days compared to AC3, and an 11–16% increase at 56 days. Notably, PMAC showed more than a 10% increase in flexural strength even after 28 days, indicating a continued improvement at longer curing ages.

The reason for these results, similar to the compressive strength findings, is likely due to the control of entrained air, which reduces overall porosity while increasing fine pores. Additionally, the smaller polymer particle size likely enabled faster penetration between the aggregate and cement, enhancing the bond strength.

This is believed to be due to the infiltration of small polymer particles into the relatively large spaces within the cement matrix, resulting in a denser comatrix structure.

(3) Bond Strength

**Figure 10:**
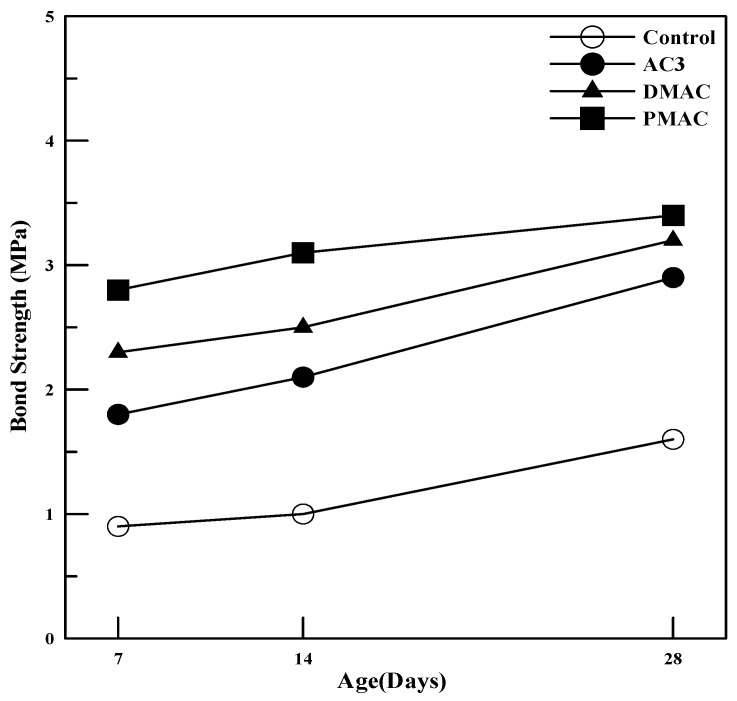
Bond strength of polymer cement mortar at different curing ages.

Figure 10 summarizes the bond strength measurements at 7, 14, and 28 days for polymer cement mortar prepared using AC3, DMAC, and PMAC, as well as ordinary cement mortar. As shown in Figure 10, the bond strength of all polymer cement mortars was 2 to 3 times higher than that of ordinary cement mortar, and bond strength increased with curing time. Additionally, DMAC and PMAC showed higher bond strength compared to AC3, with PMAC exhibiting a 20–50% increase in bond strength.

**Figure 11:**
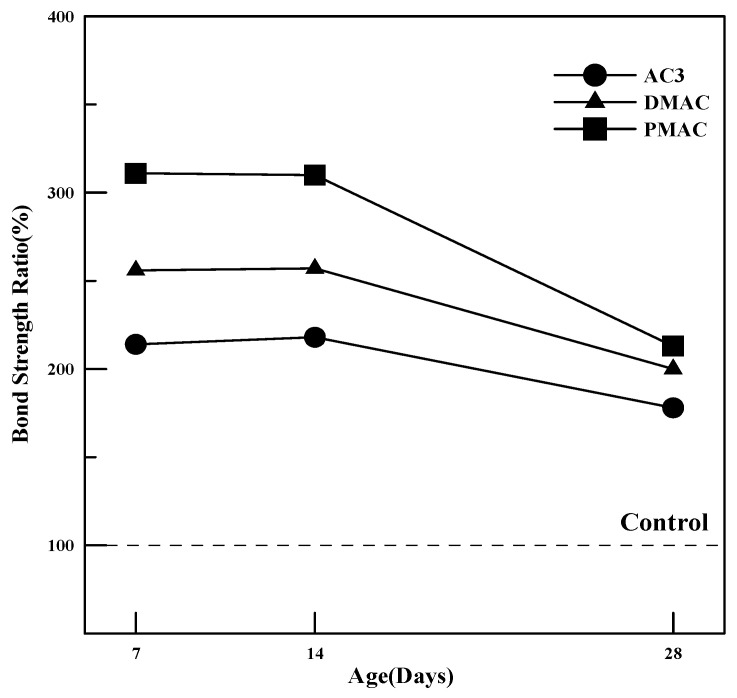
Bond strength ratio of polymer cement mortar.

To evaluate the bond strength development characteristics of the improved polymer cement mortars, bond strength was measured up to 28 days, and the bond strength ratio of polymer cement mortar to ordinary cement mortar at each curing age is summarized in Figure 11. As seen in Figure 11, the bond strength ratio at 28 days tends to be lower compared to that at 14 days.

The reason for this trend is that in ordinary cement mortar, the bond strength increases as the cement hydrates and bonds with the base concrete. Therefore, the bond strength is relatively low at early curing stages and tends to increase significantly at later stages as hydration progresses. In contrast, in polymer cement mortar, the bond is formed through the polymer film that connects the interface between the base concrete and the mortar in the early curing stages, resulting in a higher bond strength ratio at the early ages. In other words, the bond strength of cement mortar is enhanced by cement hydration, whereas, in polymer cement mortar, the polymer film serves as an adhesive.

In the case of DMAC, the reduction in internal voids due to the control of entrained air contributes to the improved bond strength. For PMAC, the smaller polymer particles quickly penetrate the interface between the base concrete and the polymer cement mortar, enhancing the bonding strength from the early curing stages.

#### 4.2.3. Volume Change

**Figure 12:**
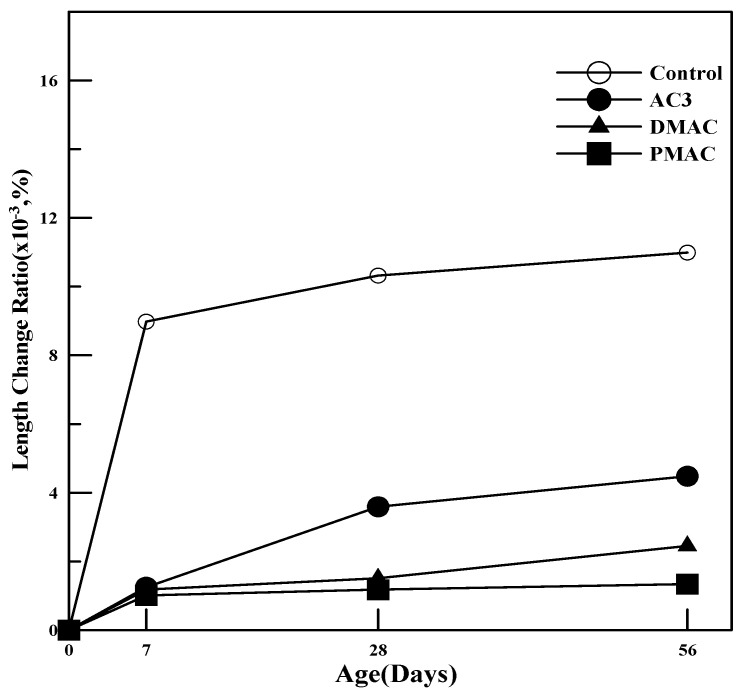
Length change rate of polymer cement mortar.

To examine the volume change of polymer cement mortar with performance-improved polymers, polymer cement mortar was prepared using three types of redispersible powder polymers: AC3, DMAC, and PMAC. The length changes up to 56 days of curing were measured according to KS F 2424 [27], and the results are shown in Figure 12.

As shown in Figure 12, the mortars incorporating the three types of powder polymers demonstrated smaller length changes compared to ordinary cement mortar, indicating reduced volume change regardless of the polymer type used. Furthermore, DMAC and PMAC, which used performance-enhanced polymers, showed improved shrinkage compared to AC3, indicating the effectiveness of polymer performance improvement.

In particular, PMAC, which was improved through modification of the polymerization method, demonstrated the smallest length change rate, indicating stable shrinkage behavior. This is likely due to the smaller polymer particle size, which allows for the rapid and dense formation of an impermeable polymer film within the polymer cement mortar. This film effectively suppresses moisture evaporation, leading to reduced drying shrinkage compared to the pre-improved polymer.

### 4.3. Water Absorption

In general, the water absorption and permeability of polymer cement mortar and concrete tend to decrease as the polymer-to-cement ratio increases, rather than being impacted by the type of polymer used [29]. To achieve polymer cement mortar and concrete with good waterproofing performance, it is important to form a continuous polymer film to create a sealing effect or fill large voids with polymer particles.

In this study, to assess the impact of redispersible powder polymers on waterproofing performance, the water absorption rates of ordinary cement mortar and polymer cement mortars using AC3, DMAC, and PMAC were measured.

**Figure 13:**
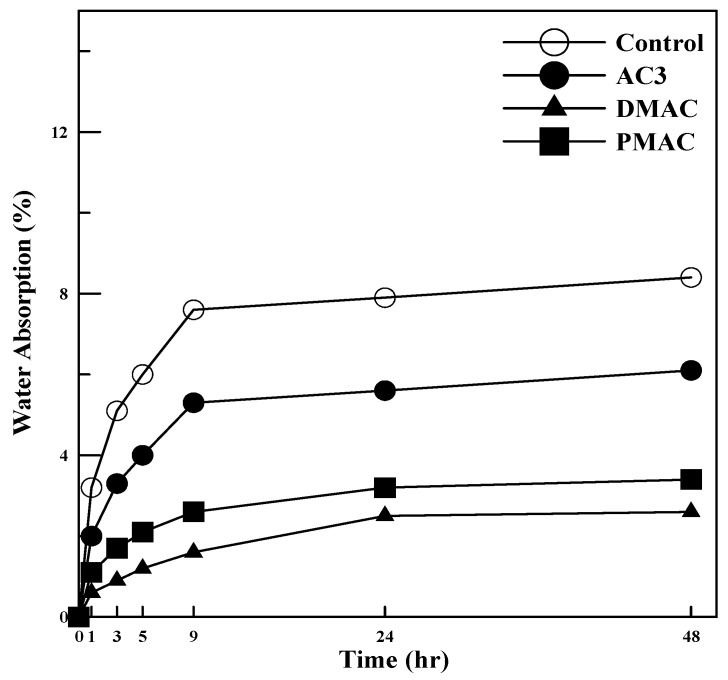
Water absorption test results.

The results of the water absorption test for polymer cement mortars using AC3, DMAC, and PMAC, as well as ordinary cement mortar, are summarized in Figure 13. As shown in Figure 13, the 48 h water absorption rate of ordinary cement mortar was 8.4%, while AC3 showed a 72.6% improvement, DMAC a 30.9% improvement, and PMAC a 40.5% improvement, indicating enhanced waterproofing performance. These results are consistent with the findings of Song Hae-Ryong et al. [30], and the observed trend is likely due to the reduced water–binder ratio, which made the structure denser, and the formation of an impermeable polymer film that prevented water absorption.

In particular, DMAC and PMAC, which were modified for improved performance, demonstrated significantly enhanced waterproofing performance compared to the pre-improvement polymers, showing that the use of functional additives and changes to the emulsion polymerization method were effective in improving water absorption resistance.

### 4.4. Microstructure Analysis

A microstructure analysis was conducted using a scanning electron microscope (SEM) and energy dispersive X-ray spectroscopy (EDS) to observe the morphology of the polymer films formed inside the polymer cement paste by the performance-modified polymers.

**Figure 14:**
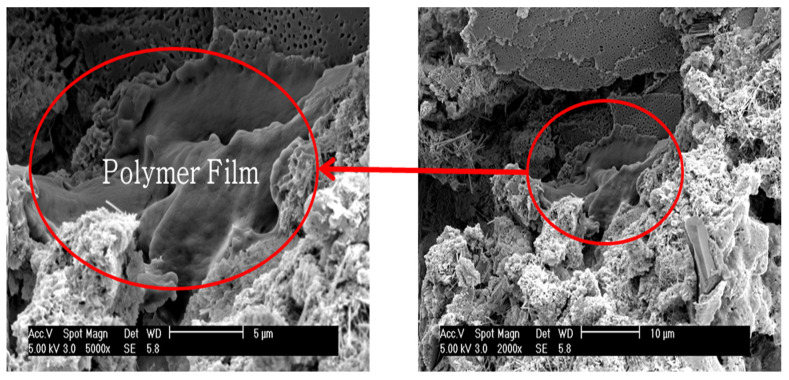
SEM image of polymer film (DMAC).

**Figure 15:**
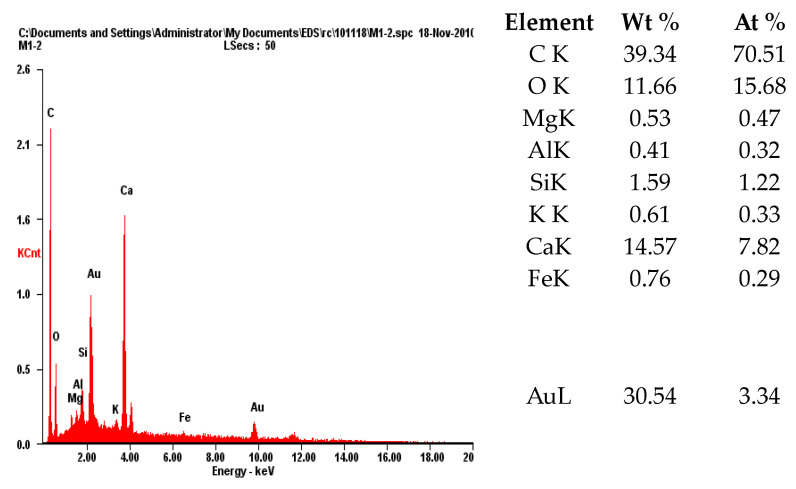
EDS analysis results of polymer film (DMAC).

**Figure 16:**
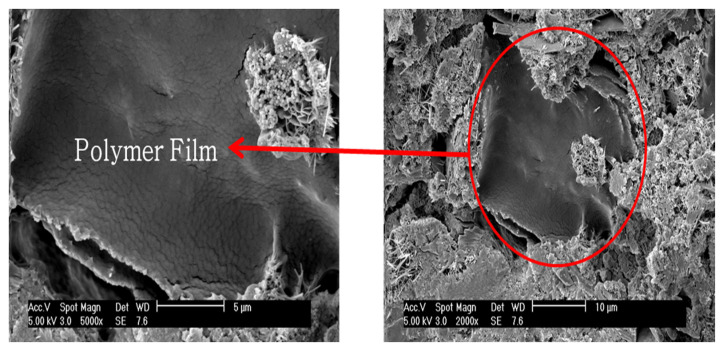
SEM image of polymer film (PMAC).

**Figure 17:**
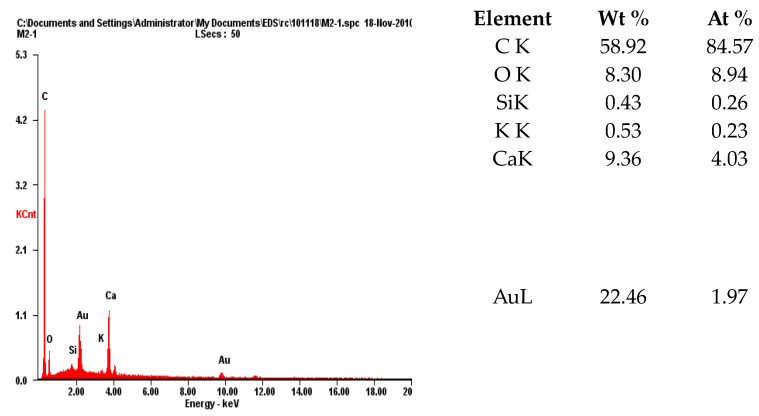
EDS analysis results of polymer film (PMAC).

Figure 14 and Figure 15 show the SEM images and EDS analysis results for the DMAC polymer film, while Figure 16 and Figure 17 show the SEM images and EDS analysis results for the PMAC polymer film. As seen in the figures, the performance-enhanced polymers penetrated the pores of the cement paste and formed films. The EDS analysis confirmed that the primary component of the formed polymer film is carbon (C).

The formed polymer film is considered to create a comatrix within the mortar, enhancing properties such as bond strength, flexural strength, and water absorption rate.

## 5. Conclusions

This study on cementitious materials using polymers modified through changes in the synthesis method and the addition of additives led to the following conclusions:

(1) In terms of tensile strength, DMAC showed higher values, while PMAC exhibited a 46% increase in elongation compared to AC3, and its average particle size decreased by approximately 35%.
(2) The compressive strength of the mortar increased by 20–40% for both DMAC and PMAC compared to AC3, and by 30–50% compared to ordinary cement mortar.
(3) The flexural strength of the mortar increased by 15–20% at 28 days for DMAC and PMAC compared to AC3, and by 11–16% at 56 days. In particular, PMAC showed more than a 10% improvement in flexural strength even after 28 days, indicating long-term strength improvement.
(4) The bond strength of all polymer cement mortars was 2–3 times higher than that of ordinary cement mortar, with bond strength increasing as the curing age progressed. DMAC and PMAC also showed improved bond strength compared to AC3, with PMAC exhibiting a 20–50% increase in strength.
(5) Compared to ordinary cement mortar, all mortars using the three types of powder polymers had smaller length change rates, indicating reduced volume change. Additionally, DMAC and PMAC, which used performance-modified polymers, showed improved shrinkage behavior compared to AC3, demonstrating the effectiveness of the polymer modifications.
(6) The water absorption test results showed that, compared to ordinary cement mortar, AC3 improved water absorption by 72.6%, DMAC by 30.9%, and PMAC by 40.5%, indicating enhanced waterproofing performance.

It was determined that the smaller the particle size of the polymer, the more effective its use, allowing for the production of a denser structure in polymer cement mortar by suppressing bubble formation. This is expected to enable effective use in repairing various types of concrete structures, such as buildings, wastewater treatment plants, bridges, dams, and roads.

## Figures and Tables

**Table 1:** Chemical composition and physical properties of Ordinary Portland Cement.

Chemical Composition		Physical Properties		
SiO_2_	21.7	Density (g/cm^2^)		3.15
Al_2_O_3_	5.7	Specific surface area (m^2^/kg)		328
Fe_2_O_3_	3.2	Setting Time (min)	Initial Set	221
CaO	63.1		Final Set	361
MgO	2.8	Compressive Strength (MPa)	3 days	22.6
SO_3_	2.2		7 days	31.6
Ig. loss	1.3		28 days	38.7

**Table 2:** Physical properties of silica sand.

Number	Size (mm)	Density (g/cm^3^)	Absorption Rate (%)	Organic Impurities
6	≤0.67	2.61	0.91	None

**Table 3:** Physical properties of silica fume.

Category	Classification	Characteristic Value
Chemical Properties (%)	SiO_2_	91.2
	Al_2_O_3_	1.3
	Fe_2_O_3_	0.8
	CaO	0.7
	MgO	0.3
	Na_2_O	0.5
	K_2_O	0.8
Physical Properties	Density (g/cm^2^)	2.2
	Specific Surface Area (m^2^/kg)	20,470

**Table 4:** Monomer composition ratio.

Category	AC3	DMAC	PMAC
Tg (°C)	−11	−11	−11
MMA (%)	45	45	45
BA (%)	30	30	30
EA (%)	5	5	5
2-EHA (%)	20	20	20

**Table 5:** Mix proportions of polymer cement mortar.

Type	Binder (Weight Ratio)			S (Weight Ratio)	SP** (B×%)	W/B* (%)	Defoamer/Polymer (%)
	C	SF	P***				
Control	1.0	-	-	2.45	0.5	54.0	-
AC3	0.9	0.5	0.5	2.45	0.5	50.0	2.0
DMAC	0.9	0.5	0.5	2.45	0.5	50.0	2.0
PMAC	0.9	0.5	0.5	2.45	0.5	50.0	2.0

B*: C + SF + P; SP**: Super Plasticizers; P***: Polymer.

**Table 6:** Characteristics of polymer powder.

Category	AC3	DMAC	PMAC
Theoretical Tg (°C)	−11	−11	−11
Measured Tg (°C)	5.64	5.64	5.64
Tensile Strength of 20% Dispersion Film (MPa)	10.5	11.0	10.5
Elongation of 20% Dispersion Film (%)	150	150	150
Average Particle Size (nm)	831	-	290

**Table 7:** Flow and air content of polymer cement mortar.

Category	Theoretical Tg (°C)	Defoamer/ Polymer (%)	W/B (%)	Flow (%)	Air Content (%)
Theoretical Tg (°C)	-	-	54.0	164	5.6
AC3	−11	2.0	50.0	160	5.2
DMAC	−11	2.0	50.0	154	4.6
PMA	−11	2.0	50.0	157	5.9

**Table 8:** Compressive strength of polymer cement mortar.

Type	Compressive Strength (MPa)			
	3 Days	7 Days	28 Days	56 Days
Control	12.9 (100)	23.0 (100)	32.4 (100)	35.8 (100)
AC3	14.4 (112)	23.7 (103)	36.7 (113)	43.3 (121)
DMAC	20.2 (157)	33.2 (144)	44.1 (136)	47.6 (133)
PMAC	17.3 (134)	28.4 (123)	40.6 (125)	48.0 (134)

The compressive strength ratio of the polymer cement mortar relative to 100 for the control (ordinary cement mortar).

**Table 9:** Flexural strength of polymer cement mortar.

Type	Flexural Strength (MPa)		Flexural Strength Ratio (%)	
	28 Days	56 Days	28 Days	56 Days
Control	6.5	7.0	100	100
AC3	9.4	10.7	145	153
DMAC	11.3	11.9	174	170
PMAC	10.8	12.4	166	177

## Data Availability

The original contributions presented in the study are included in the article, further inquiries can be directed to the corresponding author.
